# Bone morphogenetic protein-2 and leptin but not endothelin-1 induce osteochondrogenesis through increasing oxidative stress in vascular smooth muscle cells

**DOI:** 10.1186/cc12617

**Published:** 2013-06-19

**Authors:** LS do Carmo, MCC de Andrade, D de Castro Fernandes, M Liberman

**Affiliations:** 1 Albert Einstein Research and Education Institute, Morumbi, São Paulo, SP, Brazil

## Introduction

Vascular calcification is a regulated process, which associates with coronary artery disease (CAD) and occurs through an increase in transcription factor expression such as RUNX2, MSX2 and alkaline phosphatase (ALP), then inducing calcium deposition. Bone morphogenetic protein-2 (BMP2) is a potent osteochondrogenic mediator, which is expressed in CAD. Endothelin-1 (ET1) and leptin have a role in regulating inflammation and CAD. We hypothesized that BMP2, leptin or both increase ROS formation in C57BL/6 vascular smooth muscle cells (SMC), stimulating osteochondrogenic differentiation. We also investigated the effect of ET1 in SMC osteochondrogenesis. Our objectives were: to investigate ROS production in SMC after BMP2 (50 ng/ml) and/or leptin (10 ng/ml) incubation for 6 hours; and to assess osteochondrogenic gene expression and calcification of SMC stimulated with BMP2, leptin or ET1 (10 nM).

## Methods

We assessed 2-hydroxyethidium, more specific for superoxide, and ethidium which reflects hydrogen peroxide through HPLC analysis in SMC after stimulation. SMC cells were incubated with these stimuli for 48 to 96 hours and RUNX2, MSX2, ALP mRNA and protein expression were assessed using qPCR and western blotting. We quantified SMC calcification after 14 days of stimulation through Alizarin Red staining.

## Results

The results are shown as mean ± SD and were statistically significant when pHydrogen peroxide and superoxide production increased both in BMP2 and in leptin-incubated SMC (3.77 ± 0.32 and 3.26 ± 0.26) versus control (*n = *6); pBMP2 and leptin alone increased SMC calcification (1.25 ± 0.08 and 1.28 ± 0.14) versus control after 14 days (*n = *6); pET1 alone did not stimulate osteocondrogenic mRNA expression vs. control.

## Conclusion

We showed that BMP2 and leptin increased ROS formation in SMC, which stimulated osteocondrogenic mRNA/protein expression to induce SMC calcification. ET1 alone did not increase osteochondrogenesis in SMC.

